# Supplementary material for: LncRNA-Mediated Adipogenesis in Different Adipocytes
Source: Int J Mol Sci. 2022 Jul 5;23(13):7488. doi: 10.3390/ijms23137488 (PMC9267348; doi:10.3390/ijms23137488)
Supplement: Supplementary file 1 [file ijms-23-07488-s001.zip › ijms-1776943-supplementary.pdf]

Supplement Table S1
Adipogenesis and development-related lncRNAs

LncRNA	Tissue	Function	Mechanism of action	Species	Subcellular location	Year	Reference
NEAT1	White adipose tissue	Promote adipogenic differentiation	Combine with SR (serine/arginine rich) protein to increase the phosphorylation level of Clk kinase and regulate the variable cleavage of PPARγ pre-mRNA	Human	nucleus	2014	[121]
HOTAIR	White adipose tissue	Promote adipogenic differentiation	Promote the expression of adipogenic differentiation marker genes such as LPL and PPARγ; and up-regulate the expression of FABP4 and ADPN protein	Human	nucleus	2014	[136]
NEAT1	ADSCs	Promote adipogenic differentiation	miR-140 enters the nucleus and binds to NEAT1, enhancing the structural stability of NEAT1 and promoting the expression of lipogenic marker genes	Human	nucleus	2015	—

ADINR	White adipose tissue	Promote adipogenic differentiation	Change the level of histone modification of C/EBPa	Human	nucleus	2015	[91]
MEG3	ADSCs	Inhibit adipogenic differentiation	miR-140-5p sponge	Human	—	2017	[158]
MIR31HG	White adipose tissue	Promote adipogenic differentiation	Promote the histone modification of FABP4 to promote its expression	Human	Cytoplasm, nucleus	2017	[90]
HoxA-AS3	White adipose tissue	Promote adipogenic differentiation	Promote EZH2 transcription and change the level of histone modification in the promoter region of RUNX2	Human	—	2018	[92]
TINCR	ADSCs	Promote adipogenic differentiation	ceRNA,sponge miR-31-5p,C/EBP-α could bind to the promoter region of lncRNA TINCR to activate its expression	Human	—	2018	[74]
GAS5	MSCs	Inhibit adipogenic differentiation	ceRNA,sponge miR-18a	Human	—	2018	[153]
HoxA11-AS1	White adipose tissue	Promote adipogenic differentiation	Promote the expression of adipogenic differentiation	Human	—	2018	[132]

			marker genes CEBPα, DGAT2, etc.				
H19	ADSCs	Inhibit adipogenic differentiation	miR-30a sponge	Human	—	2019	—
AC092159.2	HPAV	Promote adipogenic differentiation； Promote lipid deposition	Inhibit TMEM18	Human	—	2019	—
AC092834.1	ADSCs	Inhibit adipogenic differentiation	Promote DKK1 expression and inhibit the Wnt-β-catenin pathway	Human	—	2020	[75]
MALAT1	White adipose tissue	Promote adipogenic differentiation	Regulate PPARγ expression through PPAR pathway, fatty acid metabolism and insulin signal transduction pathway	Human	Mainly located in the nucleus	2020	[122]

LINC00473	Brown adipose tissue	—	Under the action of cAMP, LINC00473 is induced and shuttled into the cytoplasm, cross-linked with mitochondria and lipid droplets.	Human	—	2020	[95]
FOXC2-AS1	White adipose tissue、Brown adipose tissue	Promote the browning of white fat；Maintain brown fat function	Autophagy signaling pathway; maintains the expression levels of UCP1 and peroxisome proliferator-activated receptors	Human	—	2020	—
lncDio3os	Brown adipose tissue	Improves brown fat activity	Maternally imprinted non-coding RNA increased methylation of the Dio3os promoter in the oocytes of obese mothers activates the transcriptional activity of Dio3 decreases the action of thyroxine T3, thereby suppressing the activity of brown fat thermogenic marker genes such as Prdm16	Human	—	2021	[101]

lncROR	Brown adipocyte;Human adipose-derived stem cells	Promote the differentiation of hADSCs to brown adipocytes	Long-stranded non-coding RNA up-regulated by adenovirus type 36 (Ad36) virus treatment in human adipose stem cells using siRNA interference with the lncROR gene significantly down-regulates the ability of human adipose stem cells to brown.	Human	—	2021	—
LYPLAL1-AS1	ADSCs	Promote adipogenic differentiation	LYPLAL1-AS1/DSP complex onhibit Wnt/Wnt/β-catenin pathway	Human	—	2021	—
lnc13728	Human adipose-derived stem cells	Promote the adipogenic differentiation of hADSCs	Interfering with the lnc13728 in hADSCs significantly downregulated the ability of MSCs to differentiate into lipids, as reflected in marker genes. Mainly, it promotes the expression of ZBED3 gene to suppress the pathway WNT/β-catenin	Human	—	2021	[76]

lncRAP2	White adipose tissue	Promote adipose tissue energy expenditure	The lncRAP2-Igf2bp2 complex enhances adipogenesis and energy expenditure by stabilizing target mRNAs. Thus, the lncRAP2-Igf2bp2 complex enhances adipogenesis and energy expenditure and is associated with susceptibility to obesity-associated diabetes	Human	Cytoplasm	2021	[84]
HOTAIR	Abdominal adipogenesis	HOTAIR overexpression using human immortalized abdominal preadipocytes	Inhibition of SLITRK4 and PITPNC1 gene expression by increasing their methylation and thus involvement in abdominal fat accumulation	Human	—	2022	—
lncXIST	Brown adipose tissue	Promote brown adipocyte differentiation	RNA-binding immunoprecipitation confirmed lncXIST binding to C/EBPα, and his partial role in promoting brown fat differentiation may be confirmed by this way.	Human	Mainly located in the nucleus	2022	[80]

lnc-RAP-n	White adipose tissue	Inhibit adipogenic differentiation	bound to PPARγ and CEBPα promoter	Mouse	—	2013	—
SRA	White adipose tissue	Promote adipogenic differentiation	Promote the phosphorylation level of IRS-1 and Akt pathway	Mouse	Cytoplasm, nucleus	2014	[120]
lnc-BATE1	Brown adipose tissue	Maintain brown fat function	Forms ribonucleoprotein complex with hnRNPU	Mouse	Cytoplasm, nucleus	2015	[93]
lnc-U90926	White adipose tissue	Inhibit adipogenic differentiation	Inhibit the promoter transcriptional activity of PPARγ2	Mouse	Mainly located in the cytoplasm	2016	[99]
uc.417	Brown adipose tissue	Promote adipogenic differentiation of brown fat	Inhibit the phosphorylation level of p38MAPK pathway	Mouse	Mainly located in the nucleus	2016	[116]
Blnc1	Brown adipose tissue	Promote adipogenic differentiation of brown fat	Forms Blnc1/hnRNPU/EBF2 ribonucleoprotein complex	Mouse&Human	Mainly located in the nucleus	2016	[83]
lnc-BATE10	Brown adipose tissue	Promote adipogenic differentiation of brown fat	As a bait molecule binds CELF1 protein, thereby releasing and promoting the expression of Pgc1a	Mouse	Cytoplasm, nucleus	2017	[77]

Paral1	White adipose tissue	Promote adipogenic differentiation	Interacts with PSPC1 and hnRNP-like binding protein 14 (RBM14) to activate PPARγ	Mouse	Mainly located in the nucleus	2017	[104]
Gm15290	White adipose tissue	Promote adipogenic differentiation	miR-27b sponge; regulates PPARγ levels	Mouse	—	2017	[87]
Adiponectin AS	White adipose tissue	Inhibit adipogenic differentiation	Forms a double-stranded complex with AdipoQ mRNA to inhibit its expression	Mouse	Cytoplasm, nucleus	2018	[82]
lnc-leptin	White adipose tissue	Inhibit adipogenic differentiation	As enhancer RNA	Mouse	—	2018	[102]
GAS5	White adipose tissue	Inhibit adipogenic differentiation	Reduce the level of miR-21a-5p and significantly reduce the mRNA and protein levels of adipogenic marker genes; act as ceRNA for miR-21a-5p and improve the expression of phosphatase	Mouse	—	2018	[41]

			and tensin homolog (PTEN)				
Plnc1	White adipose tissue	Promote adipogenic differentiation；Promote lipid deposition	Reduce the methylation level of the CpG region in the PPAR-γ2 promoter, enhance the transcriptional activity of the promoter, increase the transcription of PPAR-γ2; increase the transcriptional activity of the unmethylated PPAR-γ2 promoter	Mouse	—	2018	[100]
Bmncr	White adipose tissue	Inhibit adipogenic differentiation	Promote the formation of TAZ and RUNX2/PPARG transcription complex	Mouse	—	2018	[94]
H19	White adipose tissue, brown adipose tissue; ectopic lipid deposition	Inhibit adipogenic differentiation、lipid deposition，	miR-188 sponge; maintains the thermogenic function of brown adipocytes; inhibits the expression of paternal alleles that promote the	Mouse	Cytoplasm, nucleus	2018	[88]

		maintain brown fat function	differentiation of white adipocytes				
GM13133	Brown adipose tissue	Promote adipogenic differentiation of brown fat； Promote the browning of white fat	Activate cAMP signaling pathway	Mouse	—	2018	[115]
AK079912	Brown adipose tissue	Promote adipogenic differentiation of brown fat； Promote the browning of white fat	—	Mouse	Mainly located in the nucleus	2018	[130]
CAAlnc1	White adipose tissue	Inhibit adipogenic differentiation	Blocks the binding of HuR to C/EBPα and PPARγ	Mouse	Mainly located in the cytoplasm	2019	[79]

lnc-ORA	White adipose tissue	Promote adipogenic differentiation	Increase the mRNA and protein expression levels of cell cycle markers; regulate the DNA replication process through the PI3K/AKT/mTOR pathway	Mouse	Cytoplasm, nucleus	2019	[112]
lnc-OAD	White adipose tissue	Promote adipogenic differentiation	Increase the expression level of aP2, PPAR-γ and C/EBPa; reduce the expression of β-catenin, and inhibit cell proliferation in the MCE phase, and regulate adipogenesis through the WNT/β-catenin pathway	Mouse	—	2019	[113]
lncRNA Dreh	White adipose tissue	—	Interacts with vimentin and negatively correlates with GLUT4 expression	Mouse	—	2019	[129]
slincRAD	White adipose tissue	Promote adipogenic differentiation	Direct the methylation of promoters such as p21; direct the translocation of DNMT1 protein to the area around the nucleolus in the	Mouse	Mainly located in the nucleus	2019	[103]

			S phase, and interact with DNMT1 to participate in DNA methylation				
PGC1β-OT1	White adipose tissue	Inhibit adipogenic differentiation	Decrease the protein levels of C/EBPα, PPARγ and aP2 in cells	Mouse	Cytoplasm, nucleus	2019	—
lnc 2310069B03Rik	White adipose tissue、Brown adipose tissue	Inhibit the browning of white fat	Reduce the expression level of UCP1; reduce other genes related to the function of beige adipocytes; act as a new inhibitor of β-adrenergic receptor inducing UCP1 at the transcriptional level	Mouse	—	2019	[128]
PVT1	White adipose tissue	Promote adipogenic differentiation	Binds and interacts with STAT3	Mouse	Mainly located in the nucleus	2020	[133]
lncSAMM50	White adipose tissue	Promote adipogenic differentiation	Up-regulation of genes related to fat formation	Mouse	Mainly located in the nucleus	2021	[127]

Ctcflos	White adipose tissue	Promote the brite adipocytes thermogenic	Using variable shearing to cut out more short isoforms of Prdm16, a browning gene that plays a very important role in white fat browning	Mouse	—	2021	[125]
lncFR332443	White adipose tissue	Inhibit adipogenic differentiation	Significant inhibition of preadipocyte differentiation was achieved by unidirectional promotion of RUNX2 and phosphorylation of MAPK-p38 and MAPK-ERK1/2 expressions.	Mouse	—	2021	[117]
lncLIPE-AS1	White adipose tissue	Promote adipogenic differentiation	Down-regulation of lipogenic differentiation genes such as C/EBPβ inhibits preadipocyte differentiation	Mouse	Mainly located in the nucleus	2022	[126]
